# Carbon Costs of Constitutive and Expressed Resistance to a Non-Native Pathogen in Limber Pine

**DOI:** 10.1371/journal.pone.0162913

**Published:** 2016-10-05

**Authors:** Patrick J. Vogan, Anna W. Schoettle

**Affiliations:** 1 Mountain Studies Institute, Post Office Box 426, Silverton, Colorado 81433, United States of America; 2 Rocky Mountain Research Station, United States Department of Agriculture Forest Service, 240 West Prospect Road, Fort Collins, Colorado 80526, United States of America; US Geological Survey, UNITED STATES

## Abstract

Increasing the frequency of resistance to the non-native fungus *Cronartium ribicola* (causative agent of white pine blister rust, WPBR) in limber pine populations is a primary management objective to sustain high-elevation forest communities. However, it is not known to what extent genetic disease resistance is costly to plant growth or carbon economy. In this study, we measured growth and leaf-level physiology in (1) seedling families from seed trees that have previously been inferred to carry or not carry *Cr4*, the dominant *R* gene allele conferring complete, gene-for-gene resistance to WPBR in limber pine, and (2) populations that were and were not infected with *C*. *ribicola*. We found that, in the absence of *C*. *ribicola* exposure, there was no significant difference in carbon relations between families born from seed trees that harbor the resistance allele compared to those that lack it, either to plant growth and phenology or leaf-level photosynthetic traits. However, post-infection with *C*. *ribicola*, growth was significantly reduced in inoculation survivors expressing complete resistance compared to uninoculated seedlings. Furthermore, inoculation survivors exhibited significant increases in a suite of traits including photosynthetic rate, respiration rate, leaf N, and stomatal conductance and a decrease in photosynthetic water-use efficiency. The lack of constitutive carbon costs associated with *Cr4* resistance in non-stressed limber pine is consistent with a previous report that the *R* gene allele is not under selection in the absence of *C*. *ribicola* and suggests that host resistance may not bear a constitutive cost in pathosystems that have not coevolved. However, under challenge by *C*. *ribicola*, complete resistance to WPBR in limber pine has a significant cost to plant growth, though enhanced carbon acquisition post-infection may offset this somewhat. These costs and effects on performance further complicate predictions of this species’ response in warmer future climates in the presence of WPBR.

## Introduction

Limber pine (*Pinus flexilis* James) is a keystone species in western North America, and it functions in high elevation communities to stabilize otherwise dry, unoccupied slopes, to colonize communities soon after fire, to serve as a nurse plant for late-successional tree species and to provide food and habitat for animal species, particularly Clark’s nutcracker (*Nucifraga columbiana* Wilson)[1]. In the US and Canada, limber pine is experiencing and under threat of severe declines due to invasion of the non-native fungus *Cronartium ribicola* (JC Fisch. in Rabh.)–causative agent of the disease white pine blister rust (WPBR)–as well as mountain pine beetle (*Dendroctonus ponderosae* Hopkins), dwarf mistletoe (*Arceuthobium cyanocarpum* A Nels. ex Rydb.), and climate change [1–4]. In the province of Alberta, limber pine is listed as an endangered species [5], and in 2014 limber pine was assessed as Endangered nationally by the Committee on the Status of Endangered Wildlife in Canada (COSEWIC); it is recommended for legal listing as such under the Species at Risk Act (SARA) (http://www.cosewic.gc.ca/eng/sct5/index_e.cfm). Over the next 15 years, limber pine is expected to experience a 40% reduction in basal area in the United States [6].

An important dimension of management responses for this and other North American white pines is the proactive increasing of the number of genetically disease resistant trees on the landscape through (1) natural regeneration in populations with resistance and (2) planting disease-resistant genotypes in impacted and threatened communities [7–10]. One mechanism of disease resistance to WPBR being assessed is governed by an *R* gene–in limber pine, *Cr4* –which elicits a response consistent with a gene-for-gene, hypersensitive-like response that recognizes pathogen identity and arrests development of the disease early in its course [11]. Seed trees whose progeny segregate in a 1:1 ratio (resistant: susceptible) for the complete resistant phenotype (distinct foliar infection lesion, free of stem cankers, and premature needle shed of infected needles) after artificial inoculation with *C*. *ribicola* are inferred to be heterozygous for the dominant *Cr* allele [11–13]. If the frequency of the dominant allele is high in the native population, a segregation ratio approaching 3:1 (resistant: susceptible) can be observed in progeny as a result of the mating of a heterozygous seed tree and pollen donor; controlled crosses with commercial species have also shown the simple inheritance of the resistance trait. Four species naturally have complete resistance to *C*. *ribicola* conferred by major genes that are simply inherited: sugar pine (*P*. *lambertiana*, *Cr1*), western white pine (*P*. *monticola*, *Cr2*), southwestern white pine (*P*. *strobiformis*, *Cr3*), and limber pine (*Cr4*) [11–12]. In some areas *C*. *ribicola* has overcome *Cr1* and *Cr2*, further demonstrating that the *Cr R* gene(s) behave in the classic gene-for-gene interaction [12]. Genomics research to map and sequence the Cr *R* gene(s) is underway [14]. Currently the expression of the distinct complete resistance phenotype and its segregation within a family is accepted as an indication of the presence of the dominant *Cr* allele and is a cornerstone of the WPBR resistance program for sugar pine [12].

While the increase in the frequency of genetic resistance to this non-native disease is an important ecological and management goal, these efforts may themselves alter the physiology and even fundamental niche of the species if the resistance being propagated is associated with other facets of plant function [15]. One aspect of *R* gene function under considerable debate is the cost of *R* genes to plant fitness, either in terms of metabolic costs of protein synthesis or costs associated with other aspects of cellular function that may be linked to or induced by *R* genes, even in the absence of the corresponding pathogen [16–17]. In general, it is thought that unless there is some cost opposing selection for the R allele, the dominant *R* allele should reach fixation in a much higher proportion of species than has been observed [18–20]. In a seminal study, Bergelson and Purrington [21] determined that resistance genes in general bear an average cost of 3.5% to plant fitness, and Strauss et al. [22] found costs of resistance to herbivory in 82% of 88 studies assessed. In particular, in *Arabidopsis*, transgenic lines containing the *R* gene *RPM1* exhibited a 9% reduction in seed production relative to susceptible controls [23]. However, some studies have shown costs of individual *R* genes that are lower or effectively zero [16, 24]. It therefore appears that *R* genes may vary in their costs, with some being substantial and others non-existent [17, 25]. Given that *Cr4* complete resistance has been found at high gametic frequencies in naïve wild populations (identified through artificial inoculation progeny studies) and that the host and pathogen have not co-evolved, we hypothesize a minimal cost or a possible benefit of carrying the *Cr4* allele [11, 15].

*R* gene costs are usually reported as reductions in plant growth and/or reproductive output, and the mechanism(s) governing those reductions remain unclear. Growth reductions could be related to some other aspect of cellular function interacting with *R* gene functions or they may simply be a byproduct of linkage between *R* genes and those directly causing the observed fitness cost [21, 26–27]. While it has been suggested that respiration is not solely responsible for the reductions in growth and reproductive output observed in some species [23], there are few data addressing the carbon relations directly in uninfected plants differing in the presence and absence (inferred or confirmed through sequencing) of an *R* gene. Related traits, such as leaf N content, photosynthetic CO_2_ assimilation rate, and photosynthetic resource-use efficiencies may differ in conjunction with respiration rate, and together an assessment of a suite of related functional traits could assist in detecting and providing a mechanistic explanation for costs manifested as reduced growth and reproductive output.

In addition, further assessments can be made of sub-lethal effects of *C*. *ribicola* infection on resistant genotypes and how these may affect the performance of the resulting post-invasion pine populations. Costs to growth and seed output due to induced defense responses are common in plants [27–30] and may manifest as a surge in respiratory rate during the period of infection [31] and reduced plant growth and fitness in the months afterwards [32]. This is thought to be a product of the hypersensitive response [16], which in the white pine-WPBR pathosystem may last several months [11], longer than is typical of, for example, crop species. Therefore, white pines that survive *C*. *ribicola* infection may be more adversely affected in growth and reproductive output than is typical of other species exhibiting *R* gene resistance. In general, the sub-lethal costs of infection to plant growth and physiology have rarely been measured in perennial plants over multiple years [33], and detailed measurements of post-infection growth and carbon physiology, which are generally lacking in the white pine-WPBR pathosystem, could inform how *C*. *ribicola* invasion may shape the physiological traits of post-invasion populations through direct effects on plant growth and development after infection.

This paper presents a series of studies to assess the carbon costs of constitutive and expressed *R* gene resistance to WPBR in limber pine. To assess constitutive costs, carbon relations in the absence of *C*. *ribicola* were compared between limber pine seedling families from seed trees previously inferred to have or lack the dominant *Cr4* allele. The seed trees with and without the dominant *Cr4* allele were paired within five sites and their seeds grown in a nursery common garden for a total of nine seedling family pairs. We hypothesize that the constitutive cost of resistance in the absence of WPBR under favorable growing conditions is minimal. To examine the carbon costs of expressed complete resistance we compared pre- and post-WPBR selection seedling populations sourced from 21 sites. Bulked seed lots from each site were grown in a nursery common garden and a portion of the seedlings were inoculated with *C*. *ribicola* and a portion retained without exposure to the pathogen. We compared the carbon relations of the seedling inoculation survivors that expressed the *Cr4* complete resistance phenotype (and therefore inferred to carry the dominant *Cr4* allele) with predominantly susceptible uninoculated controls. We hypothesize that the post-WPBR inoculation populations, composed of seedlings expressing the *Cr4* resistance phenotype, will bear a cost, resulting in a significant delay in seedling development, altered carbon relations, and reduced annual growth compared to the naïve uninoculated seedling populations.

## Materials and Methods

### Seed sources and growth conditions

For the comparison of constitutive differences in carbon costs between resistant and susceptible families, seed from 9 open-pollinated seed trees previously shown to have the dominant *Cr4* allele (R families) were paired with seed from 9 open-pollinated seed trees previously shown to lack the *Cr4* allele (S families) from the same native forest sites in Northern Colorado and Southern Wyoming (Table 1) [11, 15]. The five sites ranged in latitude from 39.93 to 41.27°N and in elevation from 2370 to 3325 m (S1 Table). Progeny from these seed trees (families) were previously tested for Mendelian segregation for the complete resistance phenotype [11]; an R family is one that showed at least a 1:1 segregation of phenotypes (resistant: susceptible) after controlled inoculation with *C*. *ribicola* and therefore the source seed tree is inferred to be heterozygous for the *Cr4* allele (*Cr4cr4*)(see Table 1); an S family is one where most of the seedlings from a seed tree developed the disease after inoculation and the source seed tree is inferred to be homozygous recessive for the *Cr4* allele (c*r4*c*r4*). The performance of limber pine families in controlled inoculation trials is repeatable [11] and the same previously tested seed lots were used in the studies herein; consequently we can reliably deduce that the proportion of resistant and susceptible seedlings in each family would be similar to that of the same family when it formerly underwent inoculation (see Table 1). It is notable that not all individuals in each uninoculated R family possess the *Cr4* allele; they are open-pollinated progeny of a *Cr4cr4* seed tree and on average 46% of the seedlings in each R family did not inherit the *Cr4* allele and are rust-susceptible (see Table 1 for the proportion of seedlings with *Cr4* complete resistance phenotypes in each family), which would dilute the impact of resistant individuals on the observed trends, therefore making the reported associations herein conservative. Molecular markers to identify seedlings with the *Cr4* allele in an R family without exposure to *C*. *ribicola* are under development but not yet available [14]. S families are predominantly composed of WPBR susceptible seedlings; yet, for the families sampled here, on average 7% and up to 13% of the seedlings may be carrying the *Cr4* allele inherited via a *Cr4* pollen donor on site (see Table 1).

**Table 1:** The inferred genotypes of limber pine seed trees and the disease resistant phenotypes of their respective seedling families used in this study for the test of constitutive costs of complete resistance.

Maternal seed tree (Site-Tree ID)	Proportion of progeny displaying the *Cr4* phenotype in previous tests ^a^	Inferred seed tree genotype ^a^
CH-130R	0.31 ^b^	*Cr4cr4*
CH-134S	0.00	*cr4cr4*
CP-137R	0.65 ^b^	*Cr4cr4*
CP-141R	0.41 ^b^	*Cr4cr4*
CP-143S	0.04	*cr4cr4*
CP-144S	0.07	*cr4cr4*
EMPN6-612R	0.54 ^b^	*Cr4cr4*
EMPN6-x572R	0.41 ^b^	*Cr4cr4*
EMPN6-x591S	0.13	*cr4cr4*
EMPN6-x595S	0.10	*cr4cr4*
JEN-148R	0.91 ^c^	*Cr4cr4*
JEN-150S	0.10	*cr4cr4*
JEN-154S	0.10	*cr4cr4*
JEN-155R	0.61 ^b^	*Cr4cr4*
PHA-107R	0.50 ^b^	*Cr4cr4*
PHA-110R	0.54 ^b^	*Cr4cr4*
PHA-113S	0.09	*cr4cr4*
PHA-114S	0.00	*cr4cr4*
Average:		
R Families	0.54	
S Families	0.07	

^a^ See Schoettle et al. [11]; number of seedlings inoculated per family was 28

^b^ fits a 1:1 segregation ratio of resistant: susceptible

^c^ fits a 1:0 segregation ratio of resistant: susceptible, genotype may be *Cr4Cr4*.

Seeds for the R and S family comparison were cold stratified for 60 days, then germinated in plastic boxes in a growth chamber (25°C, 100% RH) in April, 2010 by and at USDA Forest Service Dorena Genetic Resource Center (DGRC) in Cottage Grove, OR. Upon radicle emergence, seedlings were transplanted into Ray Leach cone-tainers (164 cm^3^; Stuewe and Sons, Inc., Tangent, OR) and moved to the greenhouse. Germination and transplantation continued (15 to 20 days) until the desired sample size was reached. The common garden was a randomized complete block design. Eighteen seedlings per family were randomly assigned to each of three blocks (N = 54 total seedlings per family) and further separated within each block into rows of 6 seedlings that were randomly assigned a location within the block. In fall 2010, the seedlings were moved outside at DGRC to be exposed to seasonal temperature regimes and in early spring 2012, they were transplanted to 2310 cm^3^ “Short One” treepots (Stuewe and Sons, Tangent, OR). During the summer season plants were watered regularly.

To assess the cost of expressed complete resistance we compared carbon relations of seedlings from uninoculated bulked seed lots with inoculation survivors that expressed complete resistance from the same seed lots. Seed bulked from 18–50 (average = 26) equally-represented open-pollinated seed trees from each of 21 natural forest sites in Northern Colorado comprised our sample populations. The 21 sites ranged in latitude from 39.10 to 40.65°N and in elevation from 2680 to 3430 m. The seed was sown in April 2009 at DGRC to yield 70 seedlings per population (according to the same protocols as the R and S families above); 60 seedlings to be inoculated with *C*. *ribicola* and 10 to remain uninoculated. In September 2009, 60 seedlings per population were inoculated with *C*. *ribicola* by the DGRC staff using established DGRC protocols ([34] modified as described in [11] for limber pine) and a spore load of 7,163 sp cm^-2^. The artificial inoculation successfully challenged the seedlings; all of the 1251 inoculated seedlings except one showed symptoms of infection or disease (<0.1% possible escapes). After inoculation, all seedlings were retained in the greenhouse until they were moved outside at DGRC in early spring 2010. On average, 75% of the 60 inoculated seedlings per population died from white pine blister rust (i.e. 75% of the seedling populations were susceptible genotypes); mortality ranged from 68–82% among the 21 populations. In early spring 2012, the Inoculation Survivors (all expressing the *Cr4* complete resistance phenotype; susceptible seedlings died) and the 10 Uninoculated seedlings from each population were transplanted to 2310 cm^3^ “Short One” treepots (Stuewe and Sons, Tangent, OR). Plant culture was the same as for the R and S families described above. The pre- and post-inoculation populations differ in genotypic composition and the one-time exposure to *C*. *ribicola* may have also induced long term systemic changes in the surviving post-inoculation seedlings. The seedlings of the uninoculated naïve populations (Uninoculated seedlings) include both resistant and susceptible genotypes (on average 75% of the uninoculated seedling populations were susceptible); the seedling survivors from those same populations after the one-time controlled inoculation with *C*. *ribicola* (i.e. Inoculation Survivor seedlings) no longer include the susceptible seedlings due to WPBR-caused mortality and are now composed of seedlings that expressed the complete resistance phenotype inferred to be conferred by *Cr4*; other unknown qualitative resistance may also have been present.

### Gas exchange and leaf tissue chemistry measurements

To assess constitutive differences in plant carbon balance between R and S families, we selected nine seedlings from each of the 18 seedling families (see Table 1) and measured net CO_2_ assimilation rates (*A*_*net*_), daytime respiration rates (*R*_*day*_), stomatal conductance (*g*_*s*_) and the ratio of intercellular-to-ambient CO_2_ concentration (*C*_*i*_*/C*_*a*_) with a LI-6400XT Portable Photosynthesis System fitted with a 6400–01 CO_2_ Injector System, 6400-02B Red/Blue LED Light Source, and 2x3cm chamber with temperature controller (Li-Cor, Inc., Lincoln, NE). These measurements were conducted from July 2 to 24, 2013 between the hours of 0800 and 1400. Measurements were taken at a leaf temperature of 25°C, reference CO_2_ concentration of 400 μmol mol^-1^, saturating photosynthetic photon flux density of 1800 μE m^-2^ s^-1^ and leaf-to-air vapor pressure difference of 1.6 kPa. We estimated *R*_*day*_ as the y-intercept of a light response curve taken at 60, 40, 20 and 10 μE m^-2^ s^-1^ (after [35–36]). The rate of gross CO_2_ assimilation (*A*_*gross*_), was calculated by adding *A*_*net*_ and *R*_*day*_ for each individual. The instantaneous water-use efficiency (WUE) of each individual was calculated by dividing the net CO_2_ assimilation rate by the stomatal conductance and multiplying the result by the leaf-to-air vapor pressure difference [37]. We also randomly selected six of the measured plants from each family to measure pre-dawn leaf respiration rate (*R*_*night*_) with the LI-6400 Portable Photosynthesis System at a leaf temperature of 25°C and CO_2_ concentration of 400 μmol mol^-1^. Finally, after these measurements were complete, we randomly selected five individuals from each of five R and five S families (one R-S pair from each site) for measurements of the response of *A*_*net*_ to *C*_*i*_ (an *A/C*_*i*_ curve) and calculated for each individual the CO_2_ compensation point (Γ) and carboxylation efficiency (initial slope of the *A/C*_*i*_ curve); we also compared *A*_*net*_ at each measurement concentration of CO_2_. *A/C*_*i*_ curves were logged at delivered reference CO_2_ concentrations of 400, 300, 200, 120, 100, 80, 60 and 50 μmol mol^-1^, then raised back to 400 μmol mol^-1^ to recover their original values of *A*_*net*_ and *g*_*s*_, then measured at 600, 800 and 1200 μmol mol^-1^. One intact attached 1-year old fascicle, with its five needles splayed to minimize shading, was used for each daytime gas exchange measurement; two intact attached fascicles were inserted into the chamber for nighttime respiration measurements to increase measurement precision. Due to the 3-dimensional nature of the pine needles, all gas exchange measurements are expressed on a total leaf surface area basis which was calculated from measurements of fascicle length in the gas exchange chamber and diameter, the latter with Mitutoyo Digimatic Calipers (Mitutoyo America Corp., Aurora, IL). After gas exchange measurements, leaf samples were dried at 60°C for 48 hrs and their mass recorded. We calculated specific leaf area (SLA) as total leaf surface area divided by leaf mass.

To assess differences in carbon balance between Inoculation Survivor and Uninoculated populations, we measured *A*_*net*_, *R*_*day*_ and *R*_*night*_ in four Inoculation Survivor and four Uninoculated seedlings from each of 9 randomly-selected populations with the LI-6400 Portable Photosynthesis System between August 5 and 14, 2013, using the cuvette conditions and methods described above. During these measurements, we also recorded *g*_*s*_ and calculated *C*_*i*_*/C*_*a*_, and WUE as described above.

For both the comparisons of R and S families and of Inoculation Survivor and Uninoculated seedlings, we assessed differences in leaf tissue chemistry in November, 2013, including % leaf nitrogen, % leaf carbon and δ^13^C. For R and S families, we bulked 1-year old needle samples from 10 plants from each of the nine R and nine S families. Likewise for the Inoculation Survivor and Uninoculated seedlings, we bulked 1-year old needle samples from 10 Inoculation Survivor and 10 Uninoculated seedlings from each of 14 populations. To prepare samples for analysis of δ^13^C, samples were dried in a 60°C oven, ground using the reciprocal saw method [38], and analyzed at the Colorado State University (Fort Collins, CO) EcoCore lab using a VG Isochrom continuous flow isotope ratio mass spectrometer (Isoprime Inc., Manchester, UK) coupled to a Carlo Erba NA 1500 elemental analyzer (Milan, Italy). We analyzed the 46 samples with six random duplicates and a control sample with known isotope composition every twelve samples to document machine precision (0.2‰). To avoid possible differences in atmospheric carbon dioxide levels at the nursery from ambient (-8‰ relative to Pee Dee Belemnite PDBM standard), samples were analyzed as the carbon isotope ratio (δ^13^C), and not the standardized isotope discrimination (Δ), using the following equation:

$$\delta^{13}C=((R_{sample}))/[(R_{standard}-1)\times 1000]$$

where R = ^13^C/^12^C and units are in per mil (‰).

### Measurements of plant growth and phenology

We measured total plant heights in R and S families to ascertain constitutive differences in plant growth. Plant height was measured as the distance from the cotyledon scar on the stem to the base of the terminal bud. Plant height was also measured in Inoculation Survivor and Uninoculated seedlings from all 21 populations in the same fashion; and included measurements of annual shoot growth increment in these plants to determine effects of inoculation on the rate of seedling development. Annual shoot growth increments were determined by measuring the distance between terminal bud scars along the stem, as these indicated the end of one season’s growth. Finally, we calculated relative growth rate (RGR) of Inoculation Survivor and Uninoculated seedlings for a given year as the natural log of the plant’s total height at the end of that season minus natural log of the plant’s total height at the end of the previous season. Measurements were taken during March, 2014.

Constitutive differences in growth phenology were assessed for R and S families during the first 14 months after sowing. All seedlings were assessed for the growth beyond cotyledons during the second growing season (Spring, 2011), the Julian day at which dormancy was broken (i.e. buds began to swell and green) and terminal leader extension was initiated in 2011, the Julian day of first non-zero measurement of plant growth after initiation of terminal leader growth in 2011, and the length of terminal bud extension on June 8, 2011 (JD = 162) which was selected because it was a period of early season growth for all trees.

### Data analysis

To assess constitutive differences in traits related to gas exchange, leaf tissue chemistry, growth, and phenology, we used a paired t-test to compare family averages between pairs of R and S families from the same site using SAS statistical software (SAS Institute, Cary, NC). We also used paired t-tests to compare the gas exchange, leaf tissue chemistry and growth of Inoculation Survivor and Uninoculated seedlings, conducted as comparisons of Inoculation Survivor and Uninoculated seedlings from the population from each site of origin that was sampled.

Dissimilarity analyses were performed using a multi-response permutation procedure based on Euclidian distance according to Mielke and Berry [39]. Similarity tests were made between R and S families for (1) gas exchange traits and (2) growth and phenology traits and between Inoculation Survivor and Uninoculated families for (1) gas exchange traits and (2) annual shoot extension growth. Significant differences in this test can reflect differences in means or ranges.

## Results

### Carbon relations of R and S limber pine families in the absence of *C*. *ribicola*

There were no significant differences between R and S families in terms of plant growth and early development and phenology, which together represent an important index of plant carbon use (Table 2). When the growth and phenology traits (SLA, Total height 2013, Initial shoot elongation, 2010 Growth from cotyledons, Julian Day of the start of active growth 2011, Terminal shoot growth on June 8) were analyzed simultaneously using the multi-response permutation procedure, R and S families were again similar (P = 0.9297).

**Table 2:** Development, phenology and early growth of R and S seedling families of limber pine.

Seedling Family Type	Growth above cotyledon node in 2010 (cm)^a^	Julian day of dormancy break/active growth in 2011^b^	Initial elongation post-dormancy in 2011^c^ (cm)	Early season terminal bud extension in 2011^d^ (cm)
R	2.24 ± 0.05	119.0 ± 0.6	1.80 ± 0.03	2.11 ± 0.05
S	2.17 ± 0.06	117.5 ± 0.8	1.74 ± 0.04	2.05 ± 0.06
*P*–value	0.66	0.60	0.53	0.63

^a^ Values are means ± standard error.

^b^ The “Julian Day of dormancy break” is the day on which the seedling reached bud break.

^c^ “Initial elongation…” is the first non-zero measurement of growth after bud break.

^d^ “Early season terminal bud extension” is the length of the terminal bud on June 8, 2011. This date was selected because all the seedlings had initiated growth by this date, but about 40% had not done so on May 31, so this was clearly an important window for early seasonal growth and captures the variability between genotypes in when and to what extent they initiated growth.

**Fig 1:**
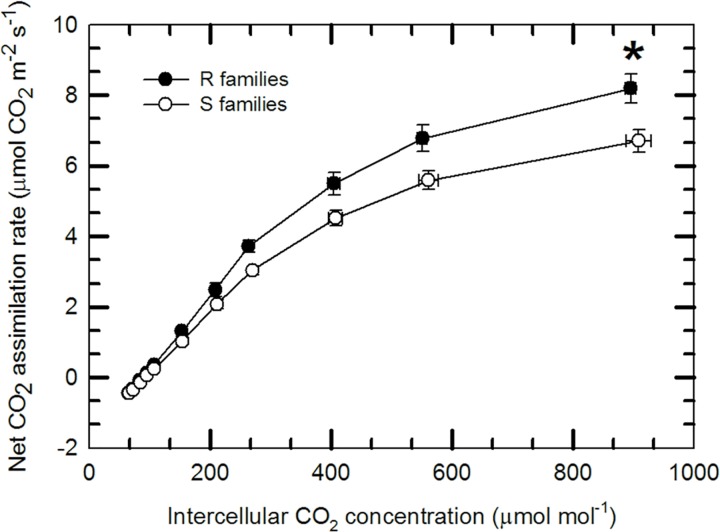
Response of net CO_2_ assimilation rate to intercellular CO_2_ concentration for R and S families of limber pine. Curves were taken at a leaf temperature of 25°C, PPFD of 1,800 μmol photons m^-2^ s^-1^ and leaf-to-air vapor pressure difference of 1.6 kPa; net CO_2_ assimilation rate is expressed on a total leaf surface area basis. Statistically significant differences are denoted by an * for P < 0.05.

Gas exchange results showed mixed differences between R and S families. Measures of day and night respiration and net CO_2_ assimilation rate were not significantly different (Table 3). However, R and S families differed significantly (*P* = 0.04) in their rate of net CO_2_ assimilation at saturating CO_2_ levels (*A*_*sat*_) (1200 μmol mol^-1^) (Fig 1). This value was 8.20 ± 0.41 μmol CO_2_ m^-2^ s^-1^ in R families and 6.71 ± 0.32 μmol CO_2_ m^-2^ s^-1^ in S families. Also, R families exhibited marginally greater rates of *A*_*net*_ (*P* ≤ 0.08) at all CO_2_ concentrations at or above current ambient levels (400 μmol mol^-1^) during *A/C*_*i*_ measurements, while values of *A*_*net*_ at CO_2_ concentrations below current ambient were not significantly different (Fig 1). When the physiological traits (*A*_*net*_, *g*_*s*_, instantaneous WUE, *C*_*i*_*/C*_*a*_, *R*_*day*_, *A*_*gross*_, *R*_*night*_, δ^13^C, % leaf N) were analyzed simultaneously using a multi-response permutation procedure [39], R and S families were similar (*P* = 0.929). The traits for the subset of R and S families for which *A/C*_*i*_ curves were measured also did not differ (previous list of traits plus carboxylation efficiency, CO_2_ compensation point, and *A*_*sat*_) (*P* = 0.9375).

**Table 3:** Parameters of leaf-level resource investment and photosynthetic resource-use for R and S families of limber pine.

Trait^a^	R families mean ± SE	S families mean ± SE	*P*-value^b^
Total plant height, Aug. 2013 (cm)	16.3 ± 0.3	16.1 ± 0.3	0.57
Net CO_2_ assimilation rate (μmol m^-2^ s^-1^)	3.65 ± 0.15	3.59 ± 0.14	0.83
Gross CO_2_ assimilation rate (μmol m^-2^ s^-1^)	3.84 ± 0.15	3.78 ± 0.15	0.84
Day respiration rate (μmol m^-2^ s^-1^)	0.166 ± 0.016	0.178 ± 0.017	0.64
Night respiration rate (μmol m^-2^ s^-1^)	0.312 ± 0.014	0.318 ± 0.015	0.77
δ^13^C (‰)	-28.4 ± 0.2	-28.3 ± 0.2	0.81
% leaf N	2.72 ± 0.12	2.51 ± 0.15	0.19
% leaf C	58.4 ± 0.6	57.7 ± 0.8	0.29
Specific leaf area (cm^-2^ g^-1^)	126.9 ± 2.4	127.0 ± 2.7	0.98
*g*_*s*_ (mol m^-2^ s^-1^)	0.044 ± 0.002	0.044 ± 0.002	0.94
Water-use efficiency (μmol mmol^-1^)	142.2 ± 3.3	142.6 ± 4.0	0.92
*C*_*i*_*/C*_*a*_ ratio	0.629 ± 0.008	0.623 ± 0.010	0.69
Carboxylation efficiency	0.0183 ± 0.0015	0.0157 ± 0.0010	0.23
CO_2_ compensation point (μmol mol^-1^)	90.3 ± 4.9	94.5 ± 4.4	0.58
*A*_*sat*_ (μmol m^-2^ s^-1^) at 1200 μmol CO_2_ mol^-1^	8.20 ± 0.41	6.71 ± 0.32	0.04

^a^ Measurements of photosynthetic gas exchange were taken at a leaf temperature of 25°C, photosynthetic photon flux density of 1800 μmol m^-2^ s^-1^, leaf-to-air vapor pressure deficit of 1.6 kPa and a reference CO_2_ concentration of 400 μmol mol^-1^, and expressed on a total leaf surface area basis.

^b^ The reported *P*-values are from a paired *t*-test comparing R and S families from the same sites.

Other traits related to photosynthetic resource-use and leaf-level resource investment were not different between R and S families when compared directly. We noted no significant differences in traits related to photosynthetic water-use, including *g*_*s*_, *C*_*i*_*/C*_*a*_ and instantaneous WUE at ambient CO_2_ concentration (Table 3). As well, both groups displayed similar values of δ^13^C, SLA and % leaf C (Table 3).

### Carbon relations of populations pre- and post-WPBR selection

**Fig 2:**
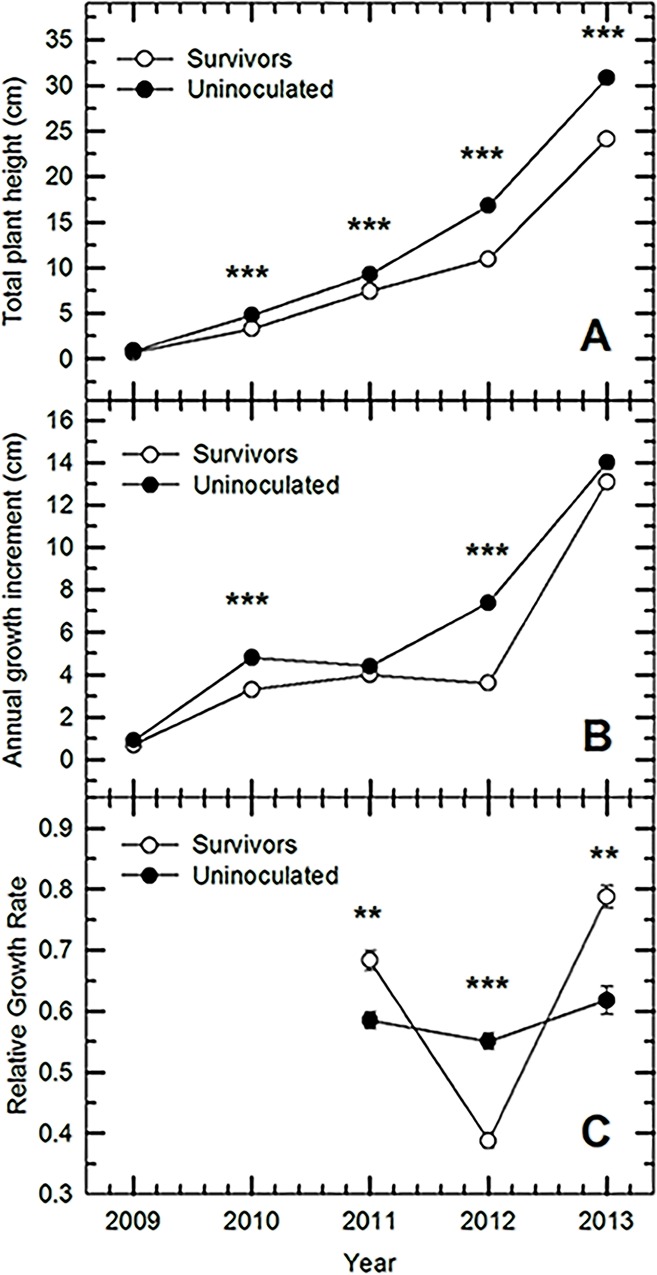
**Mean (A) total heights, (B) annual growth increments and (C) relative growth rates of Uninoculated and Inoculation Survivor bulk lots of limber pine following inoculation with *C*. *ribicola* in September, 2009.** Relative growth rate was calculated as the ln of plant height at the end of one year minus the ln of the plant’s height at the end of the previous year. Statistically significant differences are denoted as follows: *: P < 0.05, **: P < 0.01; ***: P < 0.001.

We found significant growth effects of expressed resistance to WPBR in limber pine that persisted through all four growing seasons after inoculation with *C*. *ribicola*. The most noticeable difference between Uninoculated and Inoculation Survivor seedlings was a significantly reduced total height in Inoculation Survivor seedlings each year post-inoculation (Fig 2A), especially in the years immediately following inoculation (Fig 2B). By the fourth post-inoculation growing season (2013), the Inoculation Survivors had mostly closed this gap, displaying a mean annual growth increment of 13.1 ± 0.3 cm compared to 14.0 ± 0.3 cm in Uninoculated seedlings (*P* = 0.06). Likewise, when the annual terminal extension growths for 2010, 2011, 2012, and 2013 were analyzed simultaneously, the greater growth of the Uninoculated seedlings was significantly different from Inoculation Survivors (*P*<0.0001). Relative growth rate (RGR) was significantly greater in Uninoculated seedlings only during the 2012 season; in 2011 and 2013 the Inoculation Survivor seedlings had a significantly greater RGR (Fig 2C).

Four years after inoculation (2013), the physiological traits (*A*_*net*_, *g*_*s*_, WUE, *C*_*i*_*/C*_*a*_ ratio, *R*_*day*_, *A*_*gross*_, *R*_*night*_) of Inoculation Survivor seedlings, when analyzed simultaneously using the multi-response permutation procedure, were significantly different from the physiological characteristics of Uninoculated seedlings (*P* = 0.0156)(Table 4). For the subset of populations with both physiological and leaf chemistry traits (previously listed traits plus δ^13^C, % leaf N), Inoculation Survivors were also significantly different from Uninoculated seedlings (*P* = 0.0313). In general, Inoculation Survivors had greater photosynthetic rates and were less water-use efficient than Uninoculated seedlings. When the suite of traits were tested as a whole there was a small apparent lasting effect of inoculation on these traits four years post inoculation, however there appears to be no sustained significant difference in any one leaf-level gas exchange trait in the post-inoculation Survivors and the Uninoculated seedlings (Table 4).

**Table 4:** Mean leaf-level gas exchange and resource investment parameters for Inoculation Survivor and Uninoculated populations of limber pine (± SE).

Trait^a^	Inoculation Survivors mean ± SE	Uninoculated mean ± SE	*P*-value^b^
Net CO_2_ assimilation rate (μmol m^-2^ s^-1^)	3.31 ± 0.25	2.98 ± 0.16	0.28
Gross CO_2_ assimilation rate (μmol m^-2^ s^-1^)	3.41 ± 0.26	3.09 ± 0.16	0.33
Day respiration rate (μmol m^-2^ s^-1^)	0.093 ± 0.029	0.115 ± 0.026	0.59
Night respiration rate (μmol m^-2^ s^-1^)	0.435 ± 0.024	0.494 ± 0.031	0.17
δ^13^C (‰)	-28.5 ± 0.1	-28.4 ± 0.2	0.75
% leaf N	1.96 ± 0.11	1.72 ± 0.06	0.11
% leaf C	51.8 ± 0.1	51.6 ± 0.3	0.46
Specific leaf area (cm^-2^ g^-1^)	123.2 ± 3.0	122.9 ± 2.9	0.79
*g*_*s*_ (mol m^-2^ s^-1^)	0.039 ± 0.004	0.033 ± 0.003	0.27
Water-use efficiency (μmol mmol^-1^)	152.9 ± 6.4	168.5 ± 8.0	0.17
*C*_*i*_*/C*_*a*_ ratio	0.590 ± 0.016	0.571 ± 0.020	0.47

^a^ Measurements of photosynthetic gas exchange were taken at a leaf temperature of 25°C, photosynthetic photon flux density of 1800 μmol m^-2^ s^-1^, leaf-to-air vapor pressure deficit of 1.6 kPa and a reference CO_2_ concentration of 400 μmol mol^-1^, and expressed on a total leaf surface area basis.

^b^ The reported *P*-values are from a paired *t*-test comparing Survivors and Uninoculated seedlings from the same sites.

## Discussion

### Constitutive differences in physiology between R and S families

In the absence of WPBR, the carbon relations of healthy non-stressed seedling families from seed trees that had formerly been inferred to have or lack the dominant *R* gene *Cr4* did not significantly differ in instantaneous measures of leaf-level gas exchange. We did however observe greater *A*_*sat*_ in R families, which may be indicative of greater photosynthetic capacity and carbon requirement. The elevated photosynthetic capacity at high internal CO_2_ concentrations observed here in combination with the previously reported greater cold hardiness of R families [15] suggests that R families may share traits with genotypes adapted to higher altitudes [40] compared to S families. However, the lack of a corresponding increase in leaf N makes this result difficult to interpret or be used to ascribe a possible evolutionary history of the R gene. Plant height growth, early development and phenology, which together may be influenced by carbohydrate status [41] or integrate minute differences that cannot be detected by instantaneous photosynthetic gas exchange, were also not different among R and S families in the absence of exposure to WPBR. The lack of any differences in these parameters leads us to conclude that there is no apparent differential physiological cost of *Cr4* complete resistance compared to *cr4* susceptibility in limber pine under well-watered nursery conditions in the absence of WPBR.

While limber pine is not a model system and genetic background could not be controlled, such a design can yield informative results on the potential costs of bearing an *R* gene and, perhaps, the physiological mechanism underlying it [42]. For example, Strauss et al. [22] found that 73% of studies not controlling for genetic background still detected a fitness cost of resistance. As well, environmental factors may interact with *R* genes even in model systems, potentially confounding results concerning plant fitness [16–17, 23, 43]. Sampling of paired resistant and susceptible plant families from a range of environments grown in a common garden should minimize these confounding effects to enable difference to be attributed to the difference in rust susceptibility of the family. Our lack of detection of differences between R and S families and small constitutive costs of resistance may be a consequence of other genetic variations or the reduced resolution to fully characterize the traits of resistant uninoculated seedlings due to the fact that the uninoculated R families still contain some susceptible individuals. However, differences between R and S families in A_sat_ were detected here as were differences in cold hardiness and drought responses previously [15]. *Cr4* resistance may lack a constitutive cost because this host-pathogen pathosystem has not co-evolved or some regulatory mechanism may exist in limber pine to limit the costs of particular genes depending upon biotic and abiotic environmental factors. While studies have shown significant constitutive costs of *R* genes to plant growth [23, 44], and a cost is generally expected to be present to maintain *R* gene polymorphism [45–46], the evolutionary trajectories of individual *R* genes may vary widely [47].

These results may indicate that *Cr4* is not an entirely unique gene locus, but may be an allelic variant of a similar gene in susceptible individuals that is non-functional against *C*. *ribicola*. It has been hypothesized [25] that the significant costs of *R* genes detected in some studies (e.g. [23]) are due to the insertion of a unique locus, while variation in an already present gene–particularly in the leucine-rich repeat domain, which confers pathogen specificity–may not bear a cost and may thus explain the variation in the literature on costs of *R* genes. While the *Cr4* gene in limber pine has not yet been mapped [14], recent work in sugar pine (*P*. *lambertiana*) allowed researchers to infer the presence of an ortholog to that species’ *R* gene (*Cr1*) in loblolly pine [48], which supports the hypothesis that the *Cr* genes in white pines are allelic. Whether the particular mutations producing *C*. *ribicola* specificity in *Cr4* are adaptive or neutral in the absence of the pathogen under water or freezing stress [15], there is no apparent cost to plant growth and gas exchange without environmental stress which may explain why selection has not purged the mutation(s) and how *Cr4* has persisted, sometimes at high frequencies (13.8%), in naïve limber pine forests [11]. Recent work in limber pine [11] supports this idea by revealing that *Cr4* in the southern Rockies is in equilibrium and is not under selection in mature trees in populations not yet experiencing mortality by WPBR.

### Sub-lethal effects of inoculation and post-infection population traits

There were significant effects of expressed *Cr4* complete resistance on plant growth and physiology in individuals that survived *C*. *ribicola* infection compared to seedling populations not challenged by the pathogen. Inoculation Survivor seedlings were still 21.8% shorter than Uninoculated seedlings four years after infection. It is unknown to what extent factors such as loss of leaf area [11], diversion of energy resources to plant defense [49], downregulation of photosynthetic genes in favor of defense-related genes [50], oxidative stress during a hypersensitive response [51], or some combination of these were responsible for these growth effects, but the net effect on post-infection landscape productivity may be pronounced. Given that this growth reduction was the outcome of only a single pathogen exposure event, it is reasonable to presume that repeated annual infection events *in situ* over a similar time period [33] or co-infection with another pathogen [52] may bear an even more substantial cost to plant growth, altering competitive interactions on some sites and possibly survival in harsher habitats. This impact should be considered another detrimental effect of *C*. *ribicola* on high elevation subalpine communities, even in those in which rust-resistant individuals are planted or naturally abundant.

Interestingly, while growth was significantly reduced in Inoculation Survivors immediately following inoculation, there was evidence of a compensatory growth response in the following years. Relative growth rate was significantly greater in Inoculation Survivors in two of three growing seasons post-infection, and a suite of other physiological facets of the post-infection population, including net CO_2_ assimilation rate, leaf N content and leaf respiration, were significantly greater in the Survivors. Because the population of Inoculation Survivor seedlings not only differs from the Uninoculated seedlings in the experience of inoculation but also in genotypic composition (the Inoculation Survivors contain no susceptible individuals), it is not certain if these effects were due to infection *per se*. However, since we did not observe constitutive differences between R and S families in uninfected individuals, we expect that these results are an effect of both WPBR infection and expressed complete resistance. Since a respiratory surge is not uncommon when expressing plant defenses, it is possible that enhanced photosynthetic rates and reduced WUE represent an overall acclimation towards greater carbon acquisition, which may provide more resources for plant defense and post-infection growth at the expense of plant water conservation. Compensatory growth post-infection has also been observed previously in other pathosystems [53], and this may explain the overall enhancement of leaf photosynthetic investment and growth rates after the initial infection response impact in Inoculation Survivors. R families also tend to close their stomata earlier than S families in the absence of *C*. *ribicola* when experiencing modest drought [15]; therefore, carbon demand may be even more pronounced under a combination of drought and infection, necessitating the observed increase in photosynthetic capacity. In the event that these changes are occurring in a water-limited landscape, they may further limit plant growth in a post-invasion landscape or threaten their vitality during period of water stress.

### Ecological implications

These results suggest that the proactive planting of limber pine seedlings, sown from seeds from seed trees with *Cr4* resistance, into mountain ecosystems currently uninfected with WPBR may not have a significant effect on landscape productivity and carbon economy. However, in the presence of WPBR, impacts could be substantial. Those individuals with the dominant *Cr4* allele will survive infection by wild type *C*. *ribicola* but at a significant cost to their growth and possibly their competitive abilities. While it is not certain that carbon assimilation frequently exerts a strong influence on the establishment of treeline relative to other limitations [54–57], the altered carbon relations of post-infection limber pine populations may influence the population dynamics, persistence, and migration at treeline, particularly given the importance of robust performance for success in future alpine settings [58]. Former research indicates R families are more cold hardy than S families [15] suggesting that impacts of selection for resistance on limber pine performance at treeline will likely not be a consequence of cold temperature damage. However, coniferous species adapted to cool environments can suffer from greater declines in photosynthetic rate than respiration rate under elevated temperatures [36], and the altered physiology in limber pine seedlings surviving WPBR infection may exacerbate this effect, further complicating predictions of this species’ response in warmer, and possibly also drier [59], future climates in the presence of WPBR.

Furthermore, managing for a diversity of resistance mechanisms in five-needle pine populations will provide the greatest ecosystem resilience as *C*. *ribicola* continues to spread [1, 9]. High frequencies of the *Cr* allele have occurred in small populations of western white pine (*Cr2*) and sugar pine (*Cr1*) that sustained heavy mortality by WPBR in areas of low gene flow or were artificially enriched in R gene individuals through planting, but the durability of complete resistance can be short lived; the pathogen evolved virulence (*vcr*) and the race proliferated in these areas to overcome *Cr* resistance [12]. *vcr1* appears to remain localized at sites where the frequency of *Cr1* in sugar pine tends toward fixation, while *vcr2* is more widespread and is found even in stands with low frequencies of *Cr2* [13]. In this “arms race”, where the pathogen and host coevolve, an individual R gene may be overcome and the resistant host genotypes may lose their selective advantage prior to the R allele reaching fixation in the greater host population [19]. It is unclear how this dynamic will reveal itself for *Cr4* in the limber pine–*C*. *ribicola* pathosystem. Currently, no virulence to *Cr3* or *Cr4* has been detected in *C*. *ribicola* and the open dry forests and high gene flow in these habitats may slow the proliferation of a virulent *C*. *ribicola* race [11]. Understanding the costs of *Cr4* resistance to limber pine performance helps us to understand the ecological implications of WPBR selection for this genotype as well as prepare for potential co-evolutionary scenarios in the future. Management for diverse WPBR resistance mechanisms may mitigate both the costs of *Cr4* resistance on population performance in the presence of the rust as well as reduce the proliferation of *vcr4*, if evolved.

## Conclusion

We found no significant constitutive differences in carbon economy among healthy non-stressed seedling families from seed trees with and without the *Cr4* resistance allele in limber pine. In contrast, even a one-time exposure to *C*. *ribicola* significantly reduced plant growth in surviving seedlings expressing *Cr4* complete resistance and apparently conditioned plants to have greater leaf-level investment in photosynthetic capacity and to be less water-use efficient, effects that may affect the vigor of this species and its competitive interactions in high-elevation communities in the wake of *C*. *ribicola* invasion. These factors should be taken into consideration when the management of plant communities with disease-resistant individuals is being considered and when modeling the future distribution of the species under climate change scenarios.

## Supporting Information

S1 Table Geographic characteristics of sites of origin of limber pine families in this study. (DOCX)
