# Supplementary material for: Carbon Costs of Constitutive and Expressed Resistance to a Non-Native Pathogen in Limber Pine
Source: PLoS One. 2016 Oct 5;11(10):e0162913. doi: 10.1371/journal.pone.0162913 (PMC5051957; doi:10.1371/journal.pone.0162913)
Supplement: S1 Table — (DOCX) [file pone.0162913.s001.docx]

**S1 Table. Geographic characteristics of sites of origin of limber pine families in this study.**

| **Site ID** | **Elevation (m)** | **Latitude^a^** | **Longitude^a^** |
| --- | --- | --- | --- |
| CH | 2450 | 40.96908 | -105.52701 |
| CP | 3120 | 40.65250 | -105.65515 |
| EMPN6 | 2650 | 41.25880 | -105.43317 |
| JEN | 3325 | 39.93371 | -105.65872 |
| PHA | 2660 | 41.26715 | -105.43375 |

^a^ Datum WGS84
